# Effect of Long-Term Semiarid Pasture Management on Soil Hydraulic and Thermal Properties

**DOI:** 10.3390/plants12071491

**Published:** 2023-03-29

**Authors:** Geeta Kharel, Madhav Dhakal, Sanjit K. Deb, Lindsey C. Slaughter, Catherine Simpson, Charles P. West

**Affiliations:** 1 Department of Crop, Soil and Environmental Sciences, Auburn University, Auburn, AL 36849, USA; gzk0034@auburn.edu; 2 Department of Plant and Soil Science, Texas Tech University, Lubbock, TX 79409, USA; madhav.dhakal@rodaleinstitute.org (M.D.); catherine.simpson@ttu.edu (C.S.); chuck.west@ttu.edu (C.P.W.); 3 Rodale Institute, 611 Siegfriedale Rd., Kutztown, PA 19530, USA

**Keywords:** native grass pastures, saturated hydraulic conductivity, soil water retention, thermal conductivity, soil organic matter, bulk density

## Abstract

Semiarid pasture management strategies can affect soil hydraulic and thermal properties that determine water fluxes and storage, and heat flow in unsaturated soils. We evaluated long-term (>10 years) perennial and annual semiarid pasture system effects on saturated hydraulic conductivity (*k_s_*), soil water retention curves (SWRCs), soil water thresholds (i.e., volumetric water content (θ_v_) at saturation, field capacity (FC), and permanent wilting point (PWP); plant available water (PAW)), thermal conductivity (*λ*), and diffusivity (*D_t_*) within the 0–20 cm soil depth. Forage systems included: Old World bluestem (*Bothriochloa bladhii*) + legumes (predominantly alfalfa (*Medicago sativa*)) (OWB-legume), native grass-mix (native), alfalfa + tall wheatgrass (*Thinopyrum ponticum*) (alfalfa-TW), and annual grass-mix (annual) pastures on a clay loam soil; and native, teff (*Eragrostis tef*), OWB-grazed, and OWB-ungrazed pastures on a sandy clay loam soil. The perennial OWB-legume and native pastures had increased soil organic matter (SOM) and reduced bulk density (*ρ*_b_), improving *k_s_*, soil water thresholds, *λ*, and *D_t_*, compared to annual teff and alfalfa-TW (*P* < 0.05). Soil *λ*, but not *D_t_*, increased with increasing θ_v_. Grazed pastures decreased *k_s_* and water retention compared to other treatments (*P* < 0.05), yet did not affect *λ* and *D_t_* (*P* > 0.05), likely due to higher *ρ*_b_ and contact between particles. Greater *λ* and *D_t_* at saturation and PWP in perennial versus annual pastures may be attributed to differing SOM and *ρ*_b_, and some a priori differences in soil texture. Overall, our results suggest that perennial pasture systems are more beneficial than annual systems for soil water storage and heat movement in semiarid regions.

## 1. Introduction

Soil hydraulic properties, which are needed for analyzing water fluxes and storage in unsaturated soils, can play an essential role in agricultural water management, especially in water-scarce regions [1]. Broadly, soil hydraulic properties, such as saturated hydraulic conductivity (*k*_s_) and soil water retention characteristics or curve (SWRC), are affected by both inherent and dynamic soil properties that can be managed or controlled, at least to a certain extent [2]. These soil properties include soil texture, pore size distribution, mineralogical composition, soil structure and aggregation, infiltration, bulk density (*ρ*_b_), and soil organic matter (SOM) content [3,4]. These properties can be influenced by various factors, including land uses [5,6], root biomass [7], soil fauna [8], shrinking and swelling properties of clay soils [9], freezing and thawing [10,11], and agricultural activities such as tillage [12], wheel-traffic compaction [13], and animal trampling [14].

Similarly, soil thermal properties influence the partition of energy at the soil surface and control the soil thermal regime, thereby affecting coupled liquid water, water vapor, and heat transport in unsaturated soils [15,16,17]. The fundamental soil thermal properties, such as thermal conductivity (*λ*), volumetric heat capacity (*C_v_*), and thermal diffusivity (*D_t_*), can be affected by soil texture, soil structure, mineralogical composition, aggregate size, *ρ*_b_, degree of compaction, soil porosity, soil water content, and SOM [18,19]. These properties (*λ*, *C_v_*, and *D_t_*) influence crop production systems by affecting seed germination, seedling emergence, water and nutrient uptakes by plant roots, root growth, and crop growth [20,21].

Grassland pastures are increasingly valued for their roles in conserving water and providing sustainable and economically viable alternatives to irrigated crop monocultures for the High Plains agriculture in the USA [22]. Although hydraulic and thermal properties are imperative for determining soil water fluxes, storage, and heat flow in unsaturated soils, these properties are seldom measured routinely in grassland pasture systems. This hinders our understanding of the water and thermal regimes of soil in forage production systems, which further impedes efforts to efficiently manage and conserve water resources in these semiarid pasture systems. Unsaturated soils managed under grassland and natural prairie system have shown stable pore size distribution compared to crop areas [23]. Fuentes et al. (2004) [24] reported that organic matter content in the natural prairie soil was twice the large amount seen under cultivated (i.e., conventional till and no-till) soils. The enhanced hydraulic properties were also observed in the natural prairie soil, and saturated and near-saturated hydraulic conductivities in the natural prairie were about one order of magnitude greater than in the cultivated soils [24]. Similarly, Fischer et al. (2014) [8] suggested that grasses and legumes could enhance earthworm activity, thereby influencing infiltration capacity due to changes in the burrowing activity of earthworms. Taproots of legumes can increase infiltration by forming vertical channels after decomposition [7]. Mixtures of different grass and legume species have been recommended for superior soil health [25,26] and soil water utilization [27].

Several studies have indicated ecological linkages between aboveground and belowground processes, as affected by plant community composition (e.g., microbiota assembly and nutrient mineralization), especially in C3 and C4 species [28,29]. For example, herbivory is an aboveground process that can change the quality and quantity of resources produced by plants, such as carbon and nitrogen [29], and alter soil physical properties, such as soil porosity and structure [14]. Regardless of soil texture, strategies that change SOM could impact soil water retention characteristics [30]. Besides agronomic practices, changes in land use management could alter the soil pore structures in the range from saturation (pressure head (*h*) of 0 cm) to *h* of −50 cm [31]. Schwartz et al. (2003) [5] suggested that the modifying effects of tillage, reconsolidation, and pore structure evolution on hydraulic properties are critical processes governing water movement in fine-textured soils of different land uses such as native grassland, tilled cropland, and reestablished grassland.

Studies on the effect of grassland management on soil physical properties have typically focused on variations in hydraulic conductivities due to water management, tillage, cropping systems, cover crops, soil amendments, and grazing. However, information on long-term pasture management effects on soil water retention characteristics for various annual and perennial pasture systems is limited. There is also limited information on the effect of annual and perennial pasture systems on soil water retention characteristics and their relationships to soil thermal properties. This lack of information is equally true for semiarid pasture systems with clayey soils. We considered that enhanced soil water and thermal regimes in the upper 20 cm soils were associated with improving soil physical, hydraulic, and thermal properties influenced by long-term perennial and annual pasture management practices. The objectives were to evaluate and analyze: (1) the effect of semiarid perennial and annual pasture management practices on soil hydraulic and water retention characteristics in two pasture soils with contrasting clay contents and (2) the effect of these pasture systems on soil thermal properties as a function of soil water contents given by soil water retention curves (SWRCs).

## 2. Materials and Methods

### 2.1. Study Site

**Figure 1:**
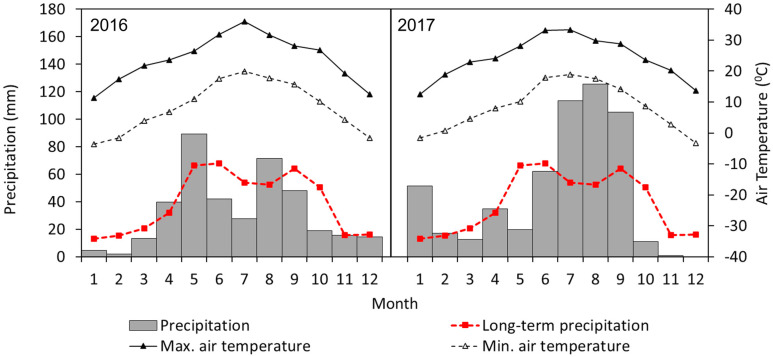
Monthly precipitation and maximum and minimum air temperatures from January 2016 to December 2017 at the study site, New Deal, TX, USA. The red color line represents long-term (1911–2008) monthly average precipitation for the study area.

The study was conducted at the Texas Tech University’s Forage-Livestock Research Laboratory Farm, New Deal, located 24 km northeast of Lubbock, TX, USA (33°43′47.2″ N, 101°43′47.1″ W at 993 m above mean sea level). The study site represented unsaturated soils that have undergone semiarid pasture management since 1997 [22]. This included variable precipitation conditions typical of the Texas Southern High Plains and various forage crop species, cattle grazing systems, water management practices (i.e., irrigation and dryland), and tillage methods. The land was nearly level, with 0 to 1% slopes. The taxonomic class of soil was Pullman clay loam (fine, mixed, superactive, thermic Torrertic Paleustolls), extensive in MLRA (Major Land Resource Area) 77C of the Southern High Plains [32]. The soil has 35 to 50% silicate clay and 0 to 3% carbonate clay content [32]. The diagnostic horizons include Ap, Bt1, Btk1, Btk2, and Btk3, with depth to secondary carbonates at 50 to 76 cm and calcic horizon at 76 to 150 cm [32]. The site has a continental, semiarid climate where the long-term mean annual precipitation for Lubbock County is 469 mm, and annual evapotranspiration is 1500 mm [33]. Monthly precipitation and air temperatures are presented in Figure 1. During the study period (2016–2017), 2016 was drier than 2017 when compared to the long-term average precipitation, especially during summer (June–September) and winter (November–December). The average daily maximum temperature exceeded 30 °C from June to August, while the minimum temperature remained below 0 °C from December to January.

### 2.2. Experiment Design and Pasture Management

**Figure 2:**
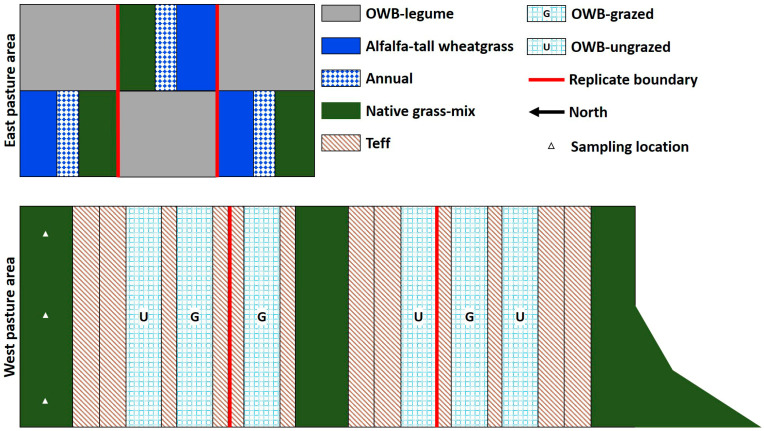
Forage crops grown in the experimental treatment plots during the 2016–2017 growing season at New Deal, TX, USA (not drawn to scale). The cultivar of Old-World bluestem (OWB) was WW-B. Dahl. Triangles in the west native grass-mix pasture represent soil sampling locations as an example for all other pastures.

The experimental site for this study had two major pasture areas, of which the east pasture area (12.3 ha) (Figure 2, top) was smaller than the west pasture (32.7 ha) (Figure 2, bottom). The establishment and management of these pastures were described by [22] and [34] (Supplementary Tables S1 and S2). The east pasture area was established in 1983 and was under perennial forages for many years (>30 years). The west pasture area, established in 2002, was previously under continuous cotton (*Gossypium hirsutum* L.) system. Treatments were only compared within, rather than between, these selected pasture areas to avoid confounding effects of time since the establishment and management history. The differences in *ρ*_b_, soil texture, and SOM between these pastures were also emphasized in previous studies [35,36]. Over the years, the establishment and management practices, such as water and irrigation management, crop rotation, use of various forage species, grazing, and tillage methods, were different for each pasture type or system in both east and west pasture areas. Different pasture systems were categorized into four broad practices (or treatments) in each pasture area to evaluate hydraulic and thermal properties in the upper 0–20 cm soil depths under long-term pasture management practices. In addition, a comparison was made between two specific pasture treatments: a grass-only (monoculture or grass-mix) and a grass-legume (two or more species) mixture.

During the study period (2016–2017), the west pasture area had pasture systems that contained either teff (*Eragrostis tef* (Zucc.) Trotter), native grass-mix (buffalograss (*Buchloe dactyloides* (Nutt.) Engelm), blue grama (*Bouteloua gracilis* (Willd. Ex Kunth) Lag. Ex Griffths), sideoats grama (*Bouteloua curtipendula* (Michx.) Torr), and green sprangletop (*Leptochloa dubia* (Kunth.) Nees)), or Old-World bluestem cv. WW-B.Dahl (OWB, *Bothriochloa bladhii* (Retz) T. Blake). The east pasture area had pasture systems containing either mixtures of OWB + alfalfa (*Medicago sativa* L.) + yellow sweetclover (*Melilotus officinalis* L.), alfalfa + tall wheatgrass (TW, *Thinopyrum ponticum* (Host) Beauv.), native, or teff (*Eragrostis tef*) (only in 2016). Since one of the replicate pastures in the east area had teff only in 2016 and was under different annual crops in previous years, such as sorghum–sudangrass (*Sorghum bicolor* (L.) Moench), wheat (*Triticum aestivum* L.), cereal rye (*Secale cereale* L.), or left fallow (annual weeds) during a severe drought in 2011 and 2012, the pasture was treated as annual. The native pastures in the east area did not have buffalograss. The grazing versus ungrazed (haying only) effect on soil hydraulic and thermal properties were evaluated for OWB pastures in the west area.

The experiment compared treatments using errors estimated with three replicate pastures nested within each pasture area (Figure 2). The east area replicates contained forage types that composed the grass-only and grass-legume systems to compare. A more favorable design would have all treatments within each spatial replicate; nevertheless, permanent physical structures of subsurface irrigation systems, fencing, and established stands did not allow the redesign of treatments and replicates. This study, aimed at evaluating the effect of perennial and annual pasture systems on soil hydraulic and thermal properties, was laid out in accordance with the layout of pasture treatments carried out over the years in the east and west pasture areas. The treatment arrangement in this study may have incurred unforeseen errors owing to a potential confounding of natural differences in soil properties between the east and west areas, for example, depth to the CaCO_3_ layer. However, the north–south placement of replicates and the separation of the east area from the west area may have minimized the potentially confounding effects on treatment differences.

### 2.3. Sample Collection and Preparation

Soil sampling was carried out from September to November in 2016 and from March to May in 2017. Soil samples were collected from three spatial points stratified in each pasture (Figure 2; see triangles in the west native treatment). Samples were taken from each end (10 m from the fence) and the middle of the pastures while maintaining a distance of at least 15 m between the sampling points. Both bulk and core soil samples were collected from each sampling point. Bulk samples were collected from 0–10 cm and 10–20 cm depths using a shovel in each treatment plot. The vegetative cover was not considered part of the surface soil sample. Collected bulk samples were stored in polyethylene zipper bags, labeled, and placed in a container to avoid evaporation. A total of 198 bulk samples (11 pastures × 3 replicates × 3 locations × 2 soil depths) were collected from the experimental site each year.

Similarly, undisturbed core samples were collected using 5 × 5 cm cylindrical cores at each sampling point. Core sampling was carried out with the help of a core sampler (with slide hammer attachment) at different soil depths down to 20 cm (i.e., 0–5, 5–10, 10–15, and 15–20 cm). Each aluminum liner with a core sample was removed from the core sampler, labeled, and sealed immediately with polyethylene end caps at both ends of the liner to prevent both soil loss and evaporation. A total of 396 soil cores (11 pastures × 3 replicates × 3 locations × 4 soil depths) were collected from the experimental site each year. All soil samples were transferred from the experimental site to the Soil Physics Laboratory at Texas Tech University. Bulk samples were air-dried, crushed, and sieved to pass a 2 mm screen before further analysis of soil properties. Bulk and core samples were stored in the refrigerator at 4 °C before analysis.

### 2.4. Determination of Soil Properties

#### 2.4.1. Basic Soil Properties

The gravimetric water content (θ_g_) of soil samples was determined from a 55 g sub-sample taken from fresh bulk samples. A pre-weighed aluminum foil was used to place the soil in a drying oven (Thermo Fisher Scientific, Waltham, MA) at 105 °C for at least 24 h until a constant dry weight was recorded. The θ_g_ (g g^−1^) was determined by dividing the weight of water by the weight of dry soil. About 50 g of air-dried sample (<2 mm) was used to conduct particle size analysis using the sedimentation (hydrometer) method [37] with the ASTM 152H hydrometer. Hydrometer readings and suspension temperature data were taken at 40 s and after 3 h, which were then used to estimate sand, clay, and silt percentages. The 5 × 5 cm core samples were used to determine soil *ρ*_b_ (g cm^−3^) by the core method [38], in which *ρ*_b_ was calculated as the ratio of the mass of dry soil solids (after oven drying at 105 °C for 24 h) to the bulk volume (volume of solids plus soil pores). The SOM content of soil samples was determined using the loss-on-ignition procedure that estimates SOM based on the gravimetric weight change associated with the high-temperature oxidation of organic matter [39]. About 10 g bulk soil sample was dried in an oven at 105 °C for 24 h and then ignited in a muffle furnace (Thomas Scientific, Swedesboro, NJ, USA) at 400 °C for 16 h. The difference in weight before (i.e., the weight of oven-dried soil at 105 °C) and after ignition (i.e., the weight of soil at 400 °C) was taken as the amount of SOM (mg g^−1^) that was present in the sample.

#### 2.4.2. Soil Hydraulic Conductivity and Soil Water Retention Characteristics

Measurements of *k*_s_ (cm d^−1^) were made on soil core samples (8 cm in diameter and 5 cm in height) by using both constant head and falling head methods [40] with a KSAT benchtop instrument (Meter Group, Inc., Pullman, WA, USA). Before *k*_s_ measurement, air-dried bulk soil samples from 0–10 and 10–20 cm depths with known *ρ*_b_ values were repacked into 8 × 5 cm sampling cylinders. The measurement of *k*_s_ was repeated three times for each core sample. In addition, a limited number of core samples using instrument-specific 8 × 5 cm cylinders were also collected from 0–10 and 10–20 cm soil depths to verify *k*_s_ measurements of saturated repacked soil cores with saturated intact cores using the KSAT instrument. Both constant head and falling head methods, which determine soil *k*_s_ using Darcy’s equation, are represented by the following governing equations (Equations (1) and (2), respectively).

(1) $$k_{s}=\frac{VL}{AtH}=\frac{QL}{AH}\left[\mathrm{where},\;Q=\frac{V}{t}\right]$$

(2) $$k_{s}=2.303\left(\frac{aL}{At_{t}}\right)\log_{10}\left(\frac{H_{0}}{H_{t}}\right)$$

where *k*_s_ is the saturated hydraulic conductivity (cm s^−1^), *V* (cm^3^) is the volume of water flowing through the cross-sectional area *A* (cm^2^) of the soil sample per unit time *t* (s), *Q* is the steady-state flow rate from Mariotte flask (cm^3^ s^−1^), *H* is the hydraulic head difference between the water inlet and outlet level (cm), *L* is the length of the soil sample (cm), *a* is the cross-sectional area of the burette (cm^2^), *H*_0_ and *H_t_* are the initial and final hydraulic head differences in the burette (cm), and *t_t_* is the time taken for the pressure head to drop from the initial to final pressure head in the burette (s).

Soil water retention characteristics were determined using the pressure chamber method [41] for all treatments in each pasture area using 5 × 5 cm core samples taken at soil depths of 0–5, 5–10, 10–15, and 15–20 for each year. A pressure plate apparatus (Soil Moisture Equipment Corp., Santa Barbara, CA, USA) was used to determine SWRC at nine pressure heads (*h*): 0 (at saturation), −100, −330, −500, −2000, −3000, −5000, −10,000, and −15,000 cm. Core samples were saturated from the bottom, weighed, and then placed on a saturated ceramic pressure plate in the pressure plate extractor. A predetermined *h* was applied for 36 to 48 h. Once the cores were equilibrated to applied *h*, cores were removed from the extractor and weighed. The same procedure was repeated with successively lower (more negative) *h* (i.e., from −100 to −15,000 cm). The ceramic plates corresponding to higher *h* (saturation to −3000 cm) and lower *h* (−5000 to −15,000 cm) were used to determine SWRCs (soil water retention curves) in the wet and dry soil water ranges, respectively. At the end of the experiment, gravimetric (θ_g_) and volumetric (θ_v_) water contents (cm^3^ cm^−3^) of the core samples were estimated from known *ρ*_b_ (g cm^−3^) of the core samples, dry weight of the core samples, and weight of the core samples at the end of each pressure head (*h*) experiment. The θ_v_ determined at each applied *h* was averaged over three replicate soil cores. The θ_v_ values were plotted against the corresponding *h* values to obtain functional relationships between θ_v_ and *h* of the soil samples, i.e., SWRCs for all treatments in the east and west pasture areas.

To quantify water flow parameters in unsaturated soils under long-term pasture management practices in both pasture areas, the van Genuchten’s SWRC model [42], which has shown its feasibility for a wide variety of soils (e.g., [43,44]), was fitted to measured SWRCs at different soil depths (i.e., 0–5, 5–10, 10–15, and 15–20 cm) for all treatments, using a nonlinear least-squares optimization program RETC [43].

(3) $$\theta_{v}\left(h\right)=\theta_{r}+\frac{\theta_{s}-\theta_{r}}{{\left[1+{\left(\alpha_{v}\left|h\right|\right)}^{n}\right]}^{\left(1-\frac{1}{n}\right)}}$$

where θ_r_ and θ_s_ are the residual and saturated θ_v_, respectively (cm^3^ cm^−3^); *h* is the pressure head (cm); and *α*_v_ (is related to the inverse of air entry pressure head; cm^−1^) and *n* (is related to pore size distribution; dimensionless) are fitting shape parameters of the SWRC. Soil water thresholds, necessary for water management in agricultural systems, were obtained from measured SWRCs at different soil depths for all treatments in each pasture area. Soil water for *h* > −15,000 cm was considered unavailable to plants. The θ_s_ (i.e., θ_v_ at saturation) provided a measure of the total porosity of soil samples. Plant available water (PAW) was determined as the difference between field capacity water content (FC; θ_v_ at *h* = −330 cm), which is the upper limit of PAW, and permanent wilting point (PWP; θ_v_ at *h* = −15,000 cm) when the plant can no longer extract water from the soil.

#### 2.4.3. Soil Thermal Properties

For all treatments in each pasture area, measurements of *λ* (W m^−1^ K^−1^), *C_v_* (MJ m^−3^ K^−1^), and *D_t_* (mm^2^ s^−1^) were made on 5 × 5 cm core samples taken at soil depths of 0–5, 5–10, 10–15, and 15–20 cm using the KD2 Pro Thermal Properties Analyzer (Meter Group, Inc., Pullman, WA, USA). The *λ*, *C_v_*, and *D_t_* values were measured simultaneously during the SWRC measurements at applied pressure heads (*h*) of 0 (at saturation), −100, −330, −500, −2000, −3000, −5000, −10,000, and −15,000 cm. The KD2 pro instrument, equipped with a dual-needle sensor (30 mm long, 1.3 mm diameter, and 6 mm spacing), works on principles of the transient line heat source analysis [45,46]. The instrument was calibrated before measuring *λ*, *C_v_*, and *D_t_* of core samples using a Delrin verification block supplied by the manufacturer.

During SWRC measurements, after the soil water equilibrium had been reached at each of the applied pressure heads, the core samples were removed from the pressure extractor, and the dual-needle sensor was inserted at the full depth of the needles into each core sample to determine *λ*, *C_v_*, and *D_t_*. During the measurement of *λ*, *C_v_*, and *D_t_*, the KD2 pro instrument applied heat for a set of heating times (*t_h_*) to one of the needles that contained a heating element. The heat was then transferred into the soil between two needles, and the temperature was measured in the measuring needle during the heating and the cooling period following heating. The resulting data were fitted to the following equations (Equations (4) and (5)) using a nonlinear least-squares procedure [47].

(4) $$\Delta T=b_{0}t+b_{1}E_{i}\left(\frac{b_{2}}{t}\right)\;\mathrm{where}\;\Delta T=\frac{4\pi \left(T-T_{0}\right)}{q}$$

(5) $$\Delta T=b_{0}t+b_{1}\left\{E_{i}\left(\frac{b_{2}}{t}\right)-Ei\left(\frac{b_{2}}{t-t_{h}}\right)\right\}$$

where Δ*T* is the temperature rise at the measuring needle; *T*_0_ is the temperature at the start of the measurement; *T* is the measured temperature; *b*_0_, *b*_1_, and *b*_2_ are the fitting parameters; *q* is the heat input at the heated needle (W m^−1^); *E_i_* is the exponential integral that is approximated using polynomials [48]; *t* is time (s); and *t_h_* is the heating time (s). Equation (4) was applied for the first *t_h_* when the heat was on, while Equation (5) was applied when the heat was off. The *λ* and *D_t_* were then computed from Equations (6) and (7), respectively, while *C_v_* was given as the ratio of *λ* and *D_t_*.

(6) $$\lambda =\frac{1}{b_{1}}$$

(7) $$D_{t}=\frac{r^{2}}{4b_{2}}$$

where *r* is the distance from the heated needle to the measuring needle.

### 2.5. Statistical Analysis

Measured data were analyzed separately for the east and west pasture areas. The generalized linear mixed model (PROC GLIMMIX) procedure in SAS version 9.4 [49] was used [50] to test the statistical significance of measured soil hydraulic and thermal properties among pasture treatments, soil depths, treatment × depth interactions, and depth × year interactions within each pasture area. Treatment differences were considered significant when ≥least significant difference (LSD) at α = 0.05. Pasture treatments were set as a fixed effect, whereas replication was set as a random effect. The repeated measurement analysis was performed for changes over the years [51], in which the denominator df (degrees of freedom) was adjusted to obtain an appropriate standard error using the Kenward–Roger method [52]. The relationship between variables was analyzed using a regression procedure (PROC REG). The outputs from the RETC model were exported to SigmaPlot 12.5 [53] to draw SWRCs for each treatment and depth.

## 3. Results and Discussion

### 3.1. Basic Soil Properties

Sand, silt, and clay contents in unsaturated soils were not affected by management practices in both east and west pasture areas (*P* > 0.05, Table 1). The east area was more clayey (i.e., clay loam according to the USDA classification) than the west (i.e., sandy clay loam). The variability in soil texture observed in pasture areas was consistent with previous studies [36,54]. SOM contents differed among treatments at 0–20 cm depth (Table 1). There was a treatment × year interaction for the east area (*P* < 0.01), while treatments did not affect SOM in the west in 2017 (*P* > 0.05). In the east pasture area, OWB-legume had the greatest SOM compared to alfalfa-TW, native, and annual (*P* < 0.01), whereas the annual had the lowest SOM (*P* < 0.01). OWB-grazed and OWB-ungrazed had consistently greater SOM than teff in 2016 (*P* < 0.05), while the native was intermediate between OWB and teff. Grazing did not affect SOM in the west OWB pasture (*P* > 0.05).

The greater SOM content at OWB pastures could be attributed to its vegetative characteristics. It has been evident that OWB-legume produced more biomass than native, alfalfa-TW, teff, and other annual grasses because of its ability to tolerate drought and longer vegetative period [34,55,56]. This suggested that more organic matter could return to the soil after the decomposition of aboveground residue and roots. Various OWB pasture systems have been reported to enhance microbial biomass [57] and soil organic C [58].

OWB pasture in the east had greater SOM than in the west (Table 1), which was most likely the result of the inclusion of legume and perennial species over more years. In a previous study conducted by Bhandari et al. (2018) [35], OWB-alfalfa pastures had greater soil organic C than native pastures in the east area, owing to a larger microbial population size. Generally, grazing animals help mix plant residues and manure into the soil, thereby increasing microbial substrate quality. However, responses of microbial biomass C and microbial community structure to grazing have yielded contrasting results in different studies (e.g., [57,59,60,61]). For instance, Acosta-Martinez et al. (2010) [57] and Wang et al. (2006) [61] observed increased microbial biomass under grazed systems. In contrast, Ingram et al. (2008) [60] reported lower microbial biomass in grazed pasture soils compared to ungrazed soils. Overall, our results indicated that OWB, a C4 perennial grass, was very effective in building SOM, while annuals and natives were less effective.

In both pasture areas, *ρ*_b_ values were lower in the upper 0–5 cm soil depth (1.41 and 1.44 g cm^−3^ for the east and west areas, respectively) than in the 5–20 cm depth (Table 1). The treatment × year interaction was not significant for both pasture areas (*P* > 0.05). In the east area, *ρ*_b_ in the 0–5 cm depth differed among pasture treatments (*P* < 0.05), whereas treatment had no effect in the deeper soil depths (*P* > 0.05). OWB-legume pasture had lower *ρ*_b_ than alfalfa-TW and annual in the upper 0–5 cm, while native had intermediate *ρ*_b_ values between OWB-legume and alfalfa-TW and annual pastures. On the contrary, *ρ*_b_ in the 0–5 cm was not affected by treatments in the west area (*P* > 0.05). Nonetheless, the treatment effect on *ρ*_b_ was observed for 5–10 and 10–15 cm depths (*P* < 0.05). Ungrazed OWB showed lower *ρ*_b_ than OWB-grazed and teff (*P* < 0.05). As in the east pasture, natives had an intermediate *ρ*_b_ value between OWB and teff in the west area. Grazing increased *ρ*_b_ in OWB pasture by 10% over OWB-ungrazed within the 5 to 15 cm soil depth in the west area, likely owing to animal trampling as SOM content was the same in both OWB-grazed and OWB-ungrazed pastures (Table 1). Animal trampling might decrease soil macroporosity (i.e., large pore spaces) and the total pore volume of soil, thereby increasing *ρ*_b_. Higher *ρ*_b_ values under annual and teff pasture systems could be attributed to wheel traffic compaction and destruction of macropores due to tillage during planting. During soil sampling, the average gravimetric water content of the top 20 cm of soil was 10% and 11% in the east and west areas, respectively (Table 1). Native in 2016 and OWB-legume in 2017 in the east area had more soil water content than alfalfa-TW and annual. During soil sampling in the west pasture area in both years, OWB-grazed and OWB-ungrazed had more soil water content than native and teff (Table 1).

### 3.2. Soil Hydraulic Properties and Water Retention Characteristics

Saturated hydraulic conductivity (*k*_s_) values were 61 and 57 cm d^−1^ at depths of 0–10 and 10–20 cm, respectively, in the west pasture area during 2016–2017. The respective *k*_s_ values for these depths in the east area were greater, i.e., 113 and 103 cm d^−1^, even though the east pasture soils had higher clay contents. Notably, the east pasture area tended to have lower *ρ*_b_ values than the west (Table 1). Hence, the most plausible explanation for differences in *k*_s_ between pasture areas was that changes in *ρ*_b_ and the resultant changes in total soil porosity under different long-term pasture management practices comprised the primary factors influencing soil *k*_s_. Total porosity (i.e., θ_v_ at saturation determined from SWRCs) and characteristics of pores in soils have been known to impact *k*_s_. In addition, changes in vegetation species could be a driving factor influencing hydraulic properties by altering total porosity, non-capillary porosity, and macro water-stable aggregates [62]. Different vegetation types have also been reported to affect *k*_s_ through root distribution and morphological characteristics, such as root biomass and distribution (e.g., [62,63,64,65]).

**Figure 3:**
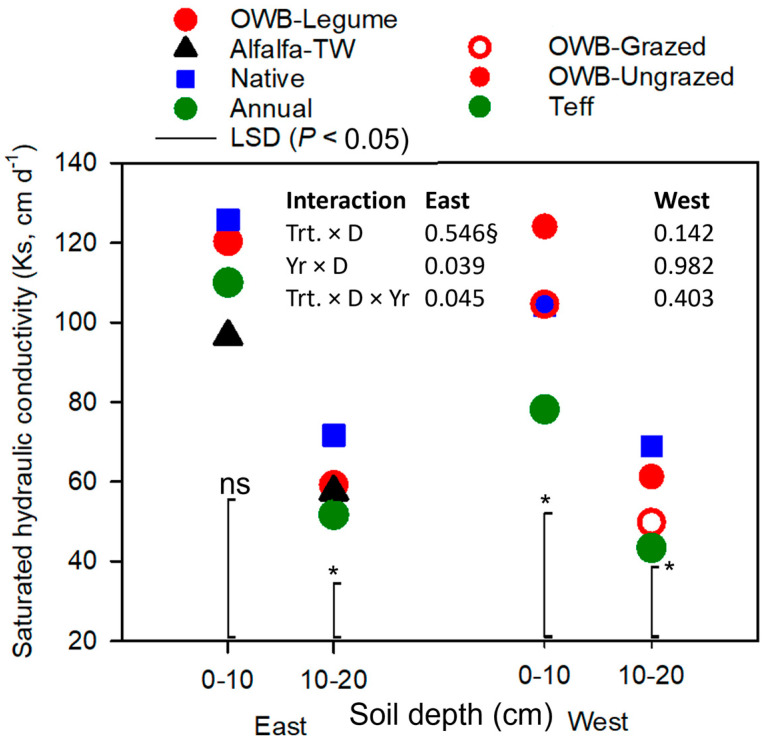
Saturated hydraulic conductivity (*k*_s_) at soil depths (D) of 0–10 and 10–20 cm for Old-World bluestem (OWB)-legume, alfalfa-tall wheatgrass (TW), native, and annual treatments (Trt.) in the east pasture area; and native, OWB-grazed, OWB-ungrazed, and teff in the west pasture area. Data were averaged across years (Yr 2016 and 2017), sampling locations, and replicates. LSD (least significant difference), represented by vertical line with the asterisk (*), is considered significant at α = 0.05; ns, LSD (vertical line without the asterisk) is nonsignificant at α = 0.05; §, *P* value of the interaction.

As shown in Figure 3, *k*_s_ in the upper 0–10 cm soil depth was not affected by pasture management in the east area (*P* > 0.05), while it was significant for the 10–20 cm depth in the east and both 0–10 and 10–20 cm depths in the west area (*P* < 0.05). In the east area, native pasture had higher *k*_s_ than annual and alfalfa-TW in the 10–20 cm depth (*P* < 0.05). For the 0–10 cm depth in the west area, OWB-ungrazed had higher *k*_s_ than teff, and teff had the lowest *k*_s_ (*P* < 0.05). Native and OWB-grazed pastures had intermediate *k*_s_ values between OWB-ungrazed and teff. In the west area, *k*_s_ in the 10–20 cm was in the order of native > OWB-ungrazed > OWB-grazed > teff. Saturated hydraulic conductivity (*k*_s_) was associated with *ρ*_b_ (r = 0.61, *P* < 0.0001) and SOM (r = 0.69, *P* < 0.0001) (data not shown) as it was greater for perennial pastures with low *ρ*_b_ and high SOM than annual. Our result was similar to previous studies, in which lower *ρ*_b_ was aligned with higher *k*_s_ (e.g., [62,66,67], and *k*_s_ tended to decrease with an increase in *ρ*_b_ with soil depth [24,62]. The finding was also consistent with previous studies focusing on perennial vegetation, in which Rachman et al. (2005) [68] and Seobi et al. (2005) [69] reported that *k*_s_ was greater under perennial vegetation than in managed annual cropland.

**Figure 4:**
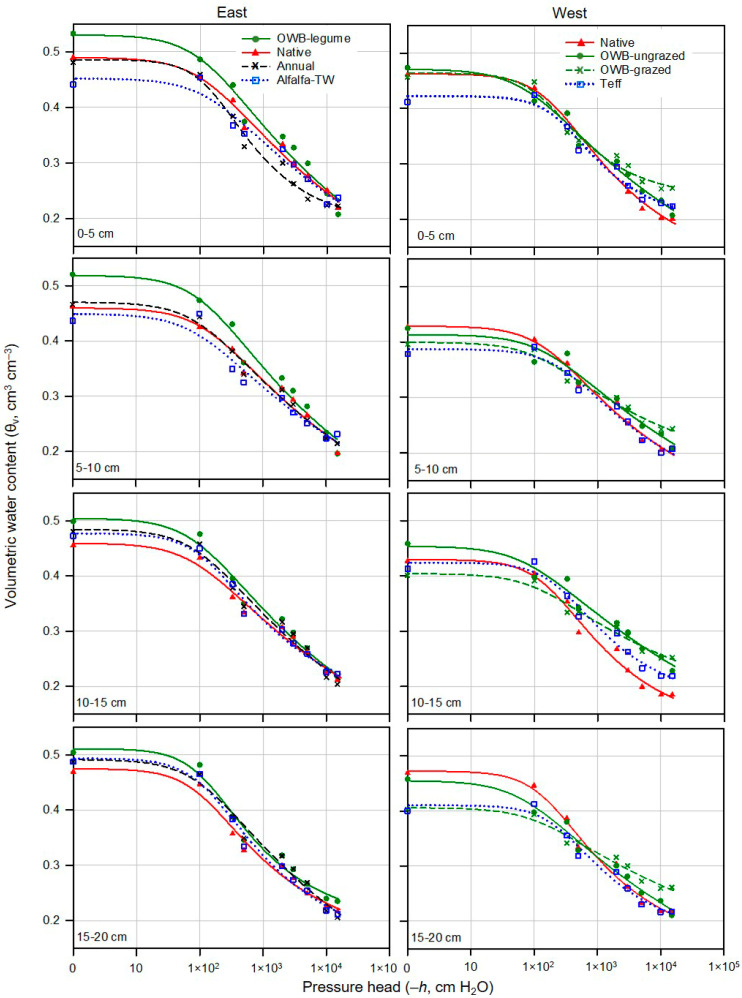
Measured (symbols) and fitted (lines) van Genuchten soil water retention curves (SWRCs) at 0–5, 5–10, 10–15, and 15–20 cm soil depths for all treatments in the east and west pasture area during a 2-year study period (2016–2017). Measured data represent average value across sampling points, replicates, and years, whereas fitted line was obtained using all sample replicates. OWB, Old-World bluestem; TW, tall wheatgrass.

The van Genuchten model was fitted to the measured SWRCs for different soil depths down to 20 cm to provide an additional tool for reliable estimates of SWRC parameters (θ_s_, θ_r_, *α_v_*, and *n*) for all treatments (Figure 4). Generally, the overall fit resulted in close agreements between measured and estimated θ_v_, as indicated by lower values of overall RMSE (root mean squared error) between 0.010 and 0.038 cm^3^ cm^−3^ and 0.013 and 0.025 cm^3^ cm^−3^ (data not shown) for the east and west pasture areas, respectively. The measured and fitted SWRCs for different pasture soils suggested that soils with greater SOM and *k*_s_ retained more water at or near saturation (i.e., air-entry region) and released less water when the *h* increased (i.e., more negative *h*), except for native and OWB-ungrazed pastures in the west area. Nonetheless, the capillary region (i.e., after air began to enter) of the SWRC was steeper with an increase in *h* for soils with greater SOM (Figure 3 and Figure 4; Table 1).

Similar to measured SWRCs at different soil depths, the patterns predicted by the modeled van Genuchten SWRCs differed among pasture treatments within and between the east and west areas (Figure 4). Accordingly, the van Genuchten parameters, especially θ_s_, *α_v_*, and *n*, showed differences as a result of pasture management practices, thereby resulting in variations in soil water thresholds (i.e., θ_v_ at saturation, FC, PWP, and PAW) in the wet and dry soil water ranges of SWRCs. Fitted van Genuchten parameter values within the 0–20 cm soil depth for all treatments in both pasture areas are listed in Table 2. The model fitting process yielded similar θ_r_ values among different treatments in both pasture areas. The residual water content (θ_r_) represents θ_v_ at the lowest *h*, at which water is retained in soil micropores [42,43,70]. The θ_r_ is often related to the amount and state of water adsorption in the mineral fractions of soil [71], which might not be affected by different pasture management practices. On the contrary, parameter *α_v_*, which determines the air entry *h* required to initiate the release of water and air begins to enter the soil pores during drainage when *h* exceeds air entry *h*, and especially parameter *n*, which describes the rate of water desorption, varied among pasture treatments within the 0–20 cm soil depths (Table 2). The variations in *α_v_* and *n* were likely due to the effect of pasture management practices on soil micropores.

Soil water thresholds, determined using SWRCs data (i.e., different soil water states from saturation to PWP) at different soil depths for each treatment, are shown in Table 3. Broadly, soil water thresholds were not affected by treatment × soil depth, year × treatment, and soil depth × year interactions at α = 0.05. An important aspect to be noted in Table 3 is that the perennial pastures, such as OWB and native, had improved soil water retention characteristics compared to annual teff (*P* < 0.05), with greater FC (0.38 to 0.44 cm^3^ cm^−3^) and PAW (0.17 to 0.23 cm^3^ cm^−3^). Generally, the east area had more water holding capacity (and PAW) than the west, which could be explained by the higher clay contents and SOM of the east pasture soils (Table 1).

It is worth noting that the type of OWB species used in this experiment (i.e., WW-B. Dahl) has been promoted for its longer vegetative period than other OWB species, which favors greater production of above- and belowground biomass [55]. Although the relation between SWRC or *k_s_* and root characteristics (e.g., distribution and morphological characteristics) was not clarified in our study, root parameters might have had effects on SWRC or *k_s_* under different pasture systems [62,72]. In the east area, θ_v_ at saturation (i.e., total soil porosity) in the 0–5 cm depth was greater for OWB-legume than alfalfa-TW and annual (*P* < 0.02), suggesting that OWB-legume treatment provided the relatively good topsoil structure.

As shown in Figure 4, when compared with native, annual, and alfalfa-TW, OWB-legume pasture retained nearly 0.05 and 0.1 cm^3^ cm^−3^ more water content in the 0–5 cm between *h* = 0 cm (at saturation) and *h* = −100 cm. Alfalfa-TW had the lowest total porosity in the 5–10 cm, where there was a parity among OWB-legume, native, and annual treatments (*P* < 0.05) (Table 3). In general, OWB-legume treatment showed greater soil water retention up to *h* of −2000 cm, while all pasture treatments, except native, had similar soil water retention characteristics after *h* of −2000 cm. Alfalfa-TW showed poor soil water retention up to *h* of −2000 cm (Figure 4). Total porosity at soil depths of 10–15 and 15–20 cm was not different among pasture treatments in the east area.

In the west pasture area, teff provided lower θ_v_ at saturation than OWB and native perennial pastures (*P* < 0.05) in the upper 0–5 cm depth (Table 3; Figure 4). As shown in Figure 4, native and both grazed and ungrazed OWB tended to have greater soil water retention in the 0–5 cm at *h* less than −100 cm. At the 5–10 cm depth, soil water retention was in the order of native > OWB-ungrazed > OWB-grazed > teff up to *h* of −330 cm (i.e., FC), but after that, native and teff released more water at an increasing rate with increasing (i.e., more negative) *h* when compared with OWB pastures. OWB-grazed delayed release of water after *h* of −1000 cm. OWB-ungrazed retained water more tightly, close to the grazed one in the 10–15 cm under *h* of −2000 cm or lower. At the 15–20 cm depth, native and OWB-ungrazed had improved soil water retention characteristics at *h* less than −100 cm than teff and OWB-grazed. Nevertheless, native and OWB-ungrazed released water under increasing *h*, while the soil impacted by grazing (OWB-grazed) held more water under the increased (i.e., more negative) *h* (Figure 4). Grazing reduced total porosity (*P* < 0.05) in the 15–20 cm, which was suggested by higher *ρ*_b_ at this 15–20 cm soil depth.

Similar effects of pasture management to those discussed for θ_v_ at saturation were observed for FC soil water threshold (i.e., θ_v_ at field capacity) (Table 3). In the east pasture area, OWB-legume and native had greater FC than alfalfa-TW (*P* < 0.05) in the 0–5 cm. Annual pasture had an intermediate FC value between native and alfalfa-TW. Native in the 5–10 cm and OWB-legume in the 10–20 cm had greater FC than alfalfa-TW (*P* < 0.05). Both native and annual treatments provided mostly similar intermediate FC values between OWB-legume and alfalfa-TW. In the west area, the effects of pasture management on FC soil water thresholds were not significant for 0–5 and 5–10 cm depths (*P* > 0.05). However, the effects of pasture management on FC for 10–15 and 15–20 cm depths were significant (*P* < 0.05). OWB-grazed treatment provided lower FC than OWB-ungrazed and native at depths of 10–20 cm and 15–20 cm, respectively (*P* < 0.05). As mentioned earlier, although soils in the west area had higher sand contents, higher *ρ*_b_ under OWB-grazed likely diminished the volume of large pores, affecting soil water retention at field capacity in denser soils.

As compared to FC soil water thresholds, an opposite trend was observed for PWP soil water threshold values under different treatments (Table 3), where pasture systems did not affect PWP in the east area (*P* > 0.05). In the west area, OWB-grazed showed greater PWP than OWB-ungrazed and native in the upper 0–5 cm (*P* < 0.05). OWB-grazed showed higher PWP compared to native pasture (*P* < 0.05) in the 0–5 cm depth (*P* < 0.05). Notably, grazing in the west area did not affect θ_v_ at PWP at soil depths of 5–10 cm and 15–20 cm (*P* > 0.05).

Following the differences observed in FC and PWP soil water thresholds at different soil depths, PAW ranged from 0.112 to 0.233 cm^3^ cm^−3^ and 0.095 to 0.190 cm^3^ cm^−3^ for the east and west pasture areas, respectively, within the 0–20 cm depth (Table 3). The east area pasture systems on clayey soils, associated with greater SOM, showed more PAW than the west. This was in alignment with prior studies (e.g., [73,74]), in which the increases in FC and PWP, as well as the greater available water content, were found for soils with higher clay and silt contents. OWB-legume had the highest PAW in the east area in the upper 0–5 cm compared to native, annual, and alfalfa-TW (*P* < 0.05). However, for the 5–10 cm depth, native pasture showed greater PAW than OWB-legume (*P* < 0.05). Alfalfa-TW treatment had PAW (0.120 cm^3^ cm^−3^), almost half of that in the OWB-legume treatment (0.229 cm^3^ cm^−3^). In the west pasture area, native and OWB-ungrazed had greater PAW than OWB-grazed in all soil depths (*P* < 0.05).

### 3.3. Soil Thermal Properties

**Figure 5:**
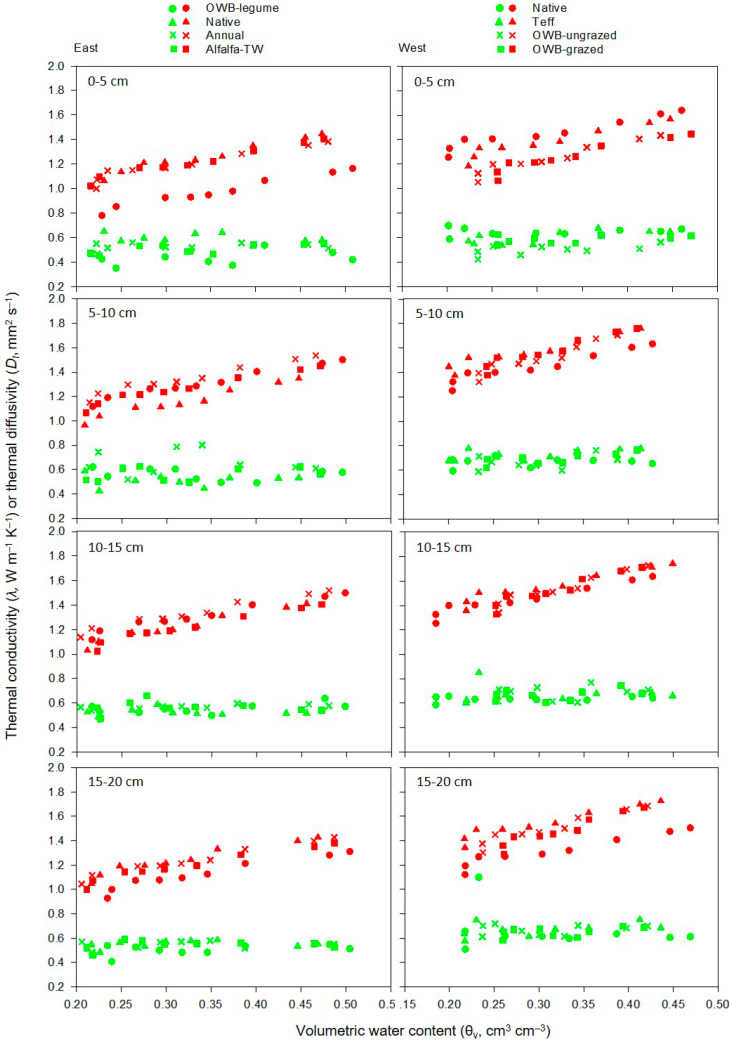
Measured soil thermal conductivity *λ* (red symbols) and thermal diffusivity *D_t_* (green symbols) as a function of volumetric water contents (θ_v_) corresponding to different applied pressure heads (i.e., 0, −100, −330, −500, −2000, −3000, −5000, −10,000, and −15,000 cm, as shown in Figure 4) at soil depths of 0–5, 5–10, 10–15, and 15–20 cm for all treatments in the east and west pasture area during a 2-year study period (2016–2017). Data were averaged across sampling points, replicates, and years. OWB, Old-World bluestem; TW, tall wheatgrass.

Soil thermal conductivity (*λ*) and diffusivity (*D_t_*) as a function of volumetric water contents (θ_v_), which correspond to applied *h* of 0 (at saturation),−100, −330, −500, −2000, −3000, −5000, −10,000, and −15,000 cm, are shown in Figure 5 for all treatments at upper soil depths from 0 to 20 cm. Soil *λ* increased with increasing θ_v_ in both pasture areas. The increase in *λ* values with increasing θ_v_ agreed with previous studies (e.g., [75]). Soil *λ* was maximum at saturation and minimum at PWP. In dry soils at PWP (<0.05 cm^3^ cm^−3^; Figure 5), as the contact points between solid particles are very small compared to the contact points between air and solid particles, heat transfer is governed by conduction within the air and by heat transfer across the gas–solid interface. Hence, *λ* of dry soils is primarily controlled by the gaseous phase and is usually low owing to the lower thermal conductivity of air than that of the other soil components (i.e., *λ*_air_ < *λ*_water_ < *λ*_mineral_). As θ_v_ increases towards saturation, the replacement of air with water continues to enhance heat conduction through the mixture because more water gathers around the contact points and forms water bridges (i.e., thermal bridges) between solid particles [76,77]. Notably, although *λ* increased with increasing θ_v_, there were no discernible changes in *D_t_* with increasing θ_v_ for all treatments in both pasture areas. The reason could be explained by very little relative changes in the *λ* and *C_v_* values of pasture soils observed in our study. The increase in *λ* with increasing θ_v_ was lower than the increase in *C_v_*. On average, *C_v_* increased by 13 and 7% when θ_v_ increased from PWP (2.33 and 2.35 MJ m^−3^ K^−1^) to saturation (2.63 and 2.51 MJ m^−3^ K^−1^) (data not shown) for the east and west pasture areas, respectively. This also suggested that while higher *λ* values ensured faster soil temperature recovery, higher *C_v_* values of pasture soils likely provided larger heat storage in the upper 0–20 cm depth.

As shown in Figure 5, *D_t_* values (as a function of θ_v_) varied between 0.39 and 0.80 mm^2^ s^−1^ and between 0.48 and 0.76 mm^2^ s^−1^ within the 0–20 cm soil depth in the east and west pasture areas, respectively. Soil *D_t_* data observed in our study were within the desirable ranges reported in previous studies [16,78,79], suggesting that both pasture soils would rapidly adjust to any temperature changes or would prevent the occurrence of temperature extremes when exposed to changes in the thermal environment. As reported by several studies (e.g., [20,80]), *λ* values in our study were also within and close to the desirable *λ* ranges that facilitated greater heat movement through the soil. For instance, Ghuman and Lal (1985) [20] found that *λ* ranged from 0.37 to 1.42 W m^−1^ K^−1^ at θ_v_ of 0.02 to 0.16 cm^3^ cm^−3^ for sandy loam, 0.35 to 3.34 W m^−1^ K^−1^ at θ_v_ of 0.02 to 0.46 cm^3^ cm^−3^ for sandy clay loam, and 0.39 to 1.15 W m^−1^ K^−1^ at θ_v_ of 0.10 to 0.52 cm^3^ cm^−3^ for clay soil, respectively.

**Figure 6:**
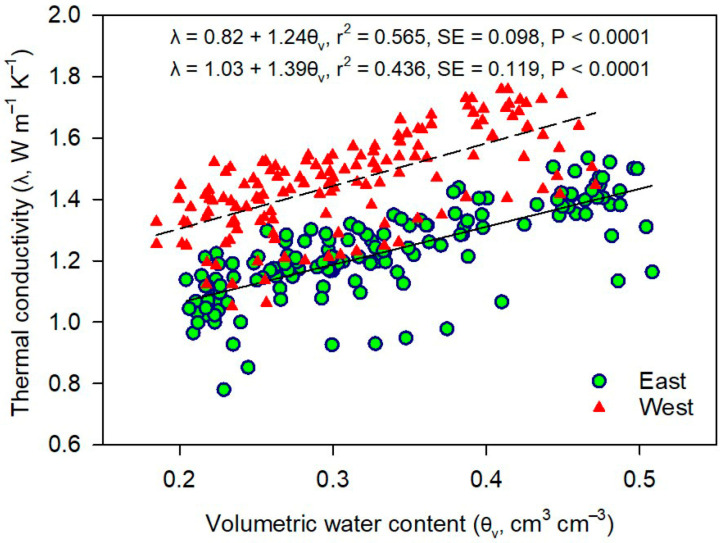
Relationship between soil thermal conductivity (*λ*) and volumetric water content (θ_v_) within the 0–20 cm soil depth for all pasture treatments in the east (solid line) and west (dashed line) area during a 2-year study period (2016–2017). The θ_v_ is a function of applied pressure heads given by measured soil water retention curves (SWRCs) (Figure 4). Data pooled from years, depths, and pressure heads applied for determining SWRCs.

The depthwise comparison of *λ* values for each pasture area showed a sudden increase in λ when θ_v_ increased from 0.20 to 0.25 cm^3^ cm^−3^, after which it increased slowly with further increases in θ_v_ up to saturation (Figure 5). The maximum *λ* of 2.03 W m^−1^ K^−1^ was observed at saturation in the 5–10 cm depth under the native pasture system. The regression between *λ* and θ_v_ (Figure 6) showed similar slopes of the linear function with a higher y-intercept for the west pasture soils than the east. In the east pasture area, native and alfalfa-TW had greater *λ* than OWB-legume at both saturation and PWP in the upper 0–5 cm depth (*P* < 0.05), while *λ* value of annual pasture soils was intermediate. Except for alfalfa-TW, the differences in *D_t_* at saturation among native, alfalfa-TW, and OWB-legume followed a similar pattern of *λ* values for the same soil depth. For the 5–20 cm depth, *D_t_* at saturation did not vary between treatments. At PWP, native had greater *D_t_* than OWB-legume and alfalfa-TW in the 0–5 cm, whereas, in the 5–10 cm depth, OWB-legume and annual had greater *D_t_* than alfalfa-TW (*P* < 0.05).

Despite having lower θ_v_ (a function of pressure heads) in the west pasture soils (Figure 4), the higher *λ* and *D_t_* in the west compared to the east could be explained by various factors, such as soil texture or particle size distribution, SOM, and *ρ*_b_ [81], which were affected by pasture management practices. Soils with higher clay contents in the east pasture (i.e., clay loam soils) likely possessed a higher degree of aggregation than soils in the west pasture (i.e., sandy clay loam soils) with greater sand contents. This suggested that poorly aggregated soils in the west area had smaller pore spaces with better contact between soil solid particles than well-aggregated soils in the east. The results agreed with previous studies, in which lower *λ* values were observed for clayey soil compared to sandy soil (e.g., [75,82,83]). The relatively lower *ρ*_b_ in the east pasture area could contribute to greater pore space, resulting in a decrease in *λ* and *D_t_* [20,83]. On the contrary, the contact between individual solid particles became more intimate for soils with the relatively higher *ρ*_b_ in the west pasture.

As discussed in Section 3.2, pasture soils with higher total porosity in the east area could decrease soil *λ* [16,84]. Organic matter, which has been reported to have greater *C_v_* and lesser *ρ*_b_ than mineral soils with a greater pore space [85,86], does not transfer heat as readily as mineral soil. Hence, it was likely that pasture soils with greater SOM in the east area could decrease *λ* and *D_t_*. Our result was in line with previous research (e.g., [20,75], [83]). For instance, Abu-Hamdeh and Reeder (2000) [75] reported a decrease in *λ* with increasing SOM, i.e., from *λ* of 0.33 W m^−1^ K^−1^ at 5% SOM to 0.17 W m^−1^ K^−1^ at 30% SOM. However, Usowicz and Lipiec (2020) [87] observed no clear trend in λ with an increasing application rate of exogenous SOM except for a few measurement dates. SOM content may indirectly affect thermal properties by altering soil aggregate structure [14], in which spherical soil aggregates tend to reduce λ and *D_t_* by changing contact areas, and by altering water storage [30], in which soil water mainly increases *λ* [76].

Based on SWRCs data (Figure 4), the variations in *λ* and *D_t_* values among different treatments were evaluated by three soil water thresholds, namely, very low θ_v_ (at PWP), intermediate θ_v_ (at FC), and high θ_v_ (at saturation). Soil *λ* and *D_t_* at saturation and PWP in different soil depths are presented in Table 4 for the east and west area. Soil *λ* and *D_t_* values in the upper 0–10 cm depth at saturation and PWP were affected by pasture treatments (*P* < 0.05), whereas *λ* and *D_t_* in the 10–20 cm depth were not affected by treatments (*P* > 0.05). Soil *λ* and *D_t_* at FC were not affected by pasture treatments (*P* > 0.05) (data not shown). Soil *λ* and *D_t_* at FC were 1.03 and 1.24 W m^−1^ K^−1^ and 0.55 and 0.66 mm^2^ s^−1^ in the east and west areas, respectively, when averaged across years and soil depths. Soil *λ* and *D_t_* values at FC were 1.31, 1.27, 1.37, and 1.31 W m^−1^ K^−1^ and 0.54, 0.53, 0.58, and 0.57 mm^2^ s^−1^ for native, OWB-legume, annual, and alfalfa-TW, respectively, in the east pasture area, while in the west area, *λ* and *D_t_* at FC were 1.51, 1.54, 1.55, and 1.60 W m^−1^ K^−1^ and 0.65, 0.67, 0.68, and 0.70 mm^2^ s^−1^ for native, OWB-ungrazed, OWB-grazed, and teff, respectively. Neither treatment × depth nor year × treatment interaction was significant for both *λ* and *D_t_* in both pasture areas (*P* > 0.05).

In the west pasture area, *λ* had no definitive trend at saturation. For example, native showed greater *λ* than OWB pastures in the 0–5 cm depth, whereas teff and OWB-grazed had greater *λ* at saturation than native in the 5–10 cm (*P* < 0.01) (Table 4). Soil *λ* under teff was higher at 0–5 and 5–10 cm depths. Pasture treatments did not affect *λ* at PWP for all soil depths (*P* > 0.05). Similarly, *D_t_* did not differ among treatments at saturation for all depths (*P* > 0.05). At PWP, only the upper 0–5 cm depth showed differences in *D_t_*, where native had greater *D_t_* than OWB-ungrazed (*P* < 0.01). Teff and OWB-grazed had intermediate *D_t_* values between native and OWB-ungrazed. For the 5–20 cm depth, *D_t_* did not vary among treatments at PWP (*P* > 0.05). Overall, greater *D_t_* within the upper 0–20 cm soil depths in the west pasture area compared to the east pasture area (Table 4) likely allowed the heat to penetrate deeper into its profile rapidly. Although heat captured at the soil surface needs to be retained to aid forage crop development, greater *D_t_* is also desirable to prevent the occurrence of temperature extremes [88], especially in semiarid environments.

## 4. Conclusions

The perennial OWB-legume and native pasture systems that contributed to the build-up of SOM and reduced *ρ*_b_ in the upper 0–20 cm soil depth significantly increased *k_s_* compared to annual (i.e., teff and alfalfa-TW) pasture systems (*P* < 0.05). The perennial pasture systems also improved soil water thresholds (i.e., θ_v_ at saturation, FC, and PWP, as well as PAW) and thermal properties (*λ* and *D_t_*) than annual pasture systems (*P* < 0.05), underscoring the benefits of permanent, perennial cover on soil water storage in semiarid environments. Native and OWB-legume pastures, especially in the clay loam east pasture, showed a similar increase in *k_s_* and soil water retention characteristics, owing to their greater SOM contents but lower *ρ*_b_ compared to annual teff and alfalfa-TW (*P* < 0.05). Soil *λ* increased with increasing θ_v_ (a function of pressure heads); however, there were no discernible changes in *D_t_* with increasing θ_v_ since the increase in *C_v_* resulted in very little relative changes in *λ* and *D_t_* of pasture soils across the range of θ_v_ given by SWRCs. Differences in greater *λ* and *D_t_* with differences in soil water thresholds between perennial and annual pastures, especially at saturation and PWP, may be attributed to differences in SOM and *ρ*_b_, in addition to a priori differences in soil texture. Grazed pastures decreased *k_s_* and soil water retention compared to other treatments (*P* < 0.05), yet did not affect *λ* and *D_t_* (*P* > 0.05), likely due to higher soil *ρ*_b_ and contact areas between particles. Significantly lower soil water retention, *k_s_*, *λ*, and *D_t_* in annual and teff pastures compared to perennial systems (*P* < 0.05) were attributed to annual disturbance, including tillage practices. Overall, our results suggest that management practices involving continued soil disturbance, including annual forage crops and tillage-based row-crop agriculture, are less beneficial to water storage and heat movement in unsaturated soils than perennial pasture systems, especially in semiarid regions.

## Figures and Tables

**Table 1:** Basic soil properties in the east and west pasture area of the experimental site. Bulk density (*ρ*_b_) and particle size distribution (soil texture) data were averaged across years. Soil texture, soil organic matter (SOM), and gravimetric water content (θ_g_) were determined in the 0–20 cm soil depth.

	East				West			
	OWB-Legume	Alfalfa-TW	Native	Annual	Native	OWB-Grazed	OWB-Ungrazed	Teff
Particle	Particle size distribution (%)							
Sand	40 (2.1) †	37 (3.0)	39 (4.2)	39 (3.7)	52 (9.2)	46 (7.6)	48 (4.1)	51 (6.0)
Silt	25 (5.5)	28 (6.9)	25 (5.1)	27 (6.4)	20 (5.4)	23 (6.3)	23 (5.1)	22 (3.5)
Clay	35 (3.8)	35 (5.3)	36 (3.8)	34 (4.4)	28 (4.2)	30 (2.8)	29 (1.0)	27 (2.5)
Year	SOM (g kg^−1^)							
2016	31 a§	19 b	27 ab	13 c	15 ab	18 a	18 a	12 b
2017	31 a	20 b	30 a	15 b	16	19	17	14
Trt. × Yr (*P* value)	0.0002				0.932			
Depth, cm	*ρ*_b_ (g cm^−3^)							
0–5	1.30 b	1.48 a	1.40 ab	1.44 a	1.48	1.45	1.38	1.44
5–10	1.50	1.43	1.46	1.54	1.52 ab	1.61 a	1.46 b	1.58 a
10–15	1.46	1.44	1.42	1.50	1.50 ab	1.54 a	1.41 b	1.55 a
15–20	1.45	1.38	1.41	1.45	1.50	1.54	1.47	1.52
Year	θ_g_ during sampling (%)							
2016	12.2 ab	9.4 b	15.1 a	9.8 b	7.8 c	14.3 ab	16.1 a	9.7 bc
2017	11.0 a	7.8 b	8.2 b	6.5 b	8.1	11.1	13.5	7.7
Trt. × Yr (*P* value)	0.0370				0.823			

† Numbers in parentheses represent standard deviation. § Means within a row followed by the same lower-case letter are not different at α = 0.05. Abbreviations: Trt., treatment; Yr, year; OWB, Old-World bluestem; TW, tall wheatgrass.

**Table 2:** Estimated van Genuchten model parameter values obtained from the simultaneous fits of the measured soil water retention curves (SWRCs) data to Equation (3) for all treatments in the east and west pasture area during a 2-year study period (2016–2017).

Soil Depth	Fitted Parameter §	East				West			
		OWB-Legume	Native	Annual	Alfalfa-TW	Native	Teff	OWB-Ungrazed	OWB-Grazed
0–5 cm	θ_r_	0.07	0.08	0.09	0.09	0.08	0.06	0.10	0.09
	θ_s_	0.54	0.48	0.48	0.44	0.46	0.42	0.47	0.46
	*α_v_*	0.06	0.08	0.11	0.12	0.01	0.01	0.01	0.03
	*n*	2.96	1.19	1.09	1.09	2.23	1.65	1.14	1.80
5–10 cm	θ_r_	0.06	0.06	0.09	0.09	0.08	0.07	0.06	0.09
	θ_s_	0.52	0.46	0.46	0.44	0.44	0.39	0.42	0.40
	*α_v_*	0.01	0.02	0.01	0.08	0.07	0.09	0.01	0.03
	*n*	1.26	1.13	1.14	2.21	1.08	1.16	1.17	1.14
10–15 cm	θ_r_	0.06	0.08	0.06	0.08	0.09	0.08	0.08	0.10
	θ_s_	0.51	0.45	0.48	0.47	0.44	0.42	0.47	0.41
	*α_v_*	0.03	0.01	0.01	0.04	0.05	0.01	0.01	0.06
	*n*	1.23	2.11	2.25	1.62	1.97	1.72	1.52	1.15
15–20 cm	θ_r_	0.05	0.06	0.07	0.06	0.06	0.07	0.07	0.08
	θ_s_	0.52	0.47	0.48	0.49	0.48	0.41	0.46	0.41
	*α_v_*	0.01	0.01	0.03	0.03	0.01	0.03	0.02	0.04
	*n*	1.56	1.42	1.15	1.15	2.14	2.42	1.54	1.12

§ θ_r_, residual volumetric water content (cm^3^ cm^−3^); θ_s_, volumetric water content at saturation (cm^3^ cm^−3^); *α*_v_ (related to the inverse of air entry pressure head; cm^−1^) and *n* (related to pore size distribution; dimensionless) are fitting shape parameters. Abbreviations: OWB, Old-World bluestem; TW, tall wheatgrass.

**Table 3:** Soil water thresholds, i.e., volumetric water contents (θ_v_) (a function of pressure heads) of pasture soils, at different soil depths for all treatments in the east and west pasture area during a 2-year study period (2016–2017). Data were averaged across sampling points, replicates, and years.

Soil Depth	East				West			
	OWB-Legume	Native	Annual	Alfalfa-TW	Native	Teff	OWB-Ungrazed	OWB-Grazed
cm	θ_v_ at saturation (cm^3^ cm^−3^)							
0–5	0.532 a§	0.489 ab	0.481 bc	0.441 c	0.460 ab	0.422 b	0.473 a	0.457 ab
5–10	0.520 a	0.463 ab	0.466 ab	0.436 b	0.437	0.388	0.424	0.396
10–15	0.513	0.452	0.480	0.471	0.437	0.423	0.468	0.408
15–20	0.522	0.474	0.484	0.487	0.479 a	0.409 b	0.458 a	0.405 b
Trt. × depth	0.999 †				0.814			
	θ_v_ at FC (cm^3^ cm^−3^)							
0–5	0.440 a	0.412 ab	0.384 bc	0.368 c	0.392	0.368	0.392	0.357
5–10	0.386 ab	0.430 a	0.382 ab	0.350 b	0.361	0.344	0.379	0.330
10–15	0.425 a	0.377 ab	0.379 ab	0.355 b	0.354 ab	0.364 ab	0.395 a	0.334 b
15–20	0.418 a	0.372 ab	0.388 ab	0.353 b	0.387 a	0.355 ab	0.380 a	0.341 b
Trt. × depth	0.874				0.906			
	θ_v_ at PWP (cm^3^ cm^−3^)							
0–5	0.223	0.219	0.207	0.238	0.201 b	0.224 ab	0.220 ab	0.256 a
5–10	0.196 b	0.197 b	0.214 ab	0.232 a	0.204	0.207	0.227	0.243
10–15	0.216	0.215	0.207	0.230	0.185 b	0.229 ab	0.242 a	0.252 a
15–20	0.238	0.215	0.216	0.224	0.217	0.217	0.221	0.261
Trt. × depth	0.104				0.768			
	PAW (cm^3^ cm^−3^)							
0–5	0.217 a	0.193 ab	0.177 b	0.130 c	0.191 a	0.144 bc	0.172 ab	0.101 c
5–10	0.190 b	0.233 a	0.168 bc	0.118 c	0.157 a	0.137 ab	0.152 a	0.087 b
10–15	0.209 a	0.162 b	0.172 ab	0.125 c	0.169 a	0.135 ab	0.153 a	0.092 b
15–20	0.180 a	0.157 ab	0.172 a	0.129 b	0.169 a	0.138 ab	0.159 a	0.080 b
Trt. × depth	0.811				0.956			

§ Means within a row followed by the same lower-case letter are not different at α = 0.05. † *P*-value of the treatment by depth interaction. Abbreviations: Trt., treatment; OWB, Old-World bluestem; TW, tall wheatgrass; PWP, permanent wilting point; FC, field capacity; PAW, plant available water.

**Table 4:** Soil thermal conductivity (*λ*) and thermal diffusivity (*D_t_*) at saturation and permanent wilting point (PWP) soil water thresholds obtained from the soil water retention curves (SWRCs) measured at different soil depths in pasture systems with OWB-legume, native grasses, annuals, and alfalfa-tall wheatgrass for the east and west area during 2016–2017.

Soil Depth	East				West			
	OWB-Legume	Native	Annual	Alfalfa-TW	Native	Teff	OWB-Ungrazed	OWB-Grazed
cm	*λ* (W m^−1^ K^−1^) at saturation							
0–5	1.16 b§	1.45 a	1.38 ab	1.41 a	1.64 a	1.57 ab	1.43 b	1.45 b
5–10	1.50	1.35	1.53	1.45	1.63 b	1.76 a	1.68 ab	1.76 a
10–15	1.50	1.41	1.52	1.41	1.64	1.74	1.73	1.71
15–20	1.31	1.43	1.43	1.38	1.50	1.73	1.69	1.67
Trt × depth	0.149				0.877			
	*λ* (W m^−1^ K^−1^) at PWP							
0–5	0.78 b	1.06 a	1.00 ab	1.02 a	1.26	1.18	1.05	1.06
5–10	1.12	0.96	1.15	1.07	1.25	1.38	1.32	1.38
10–15	1.12	1.03	1.14	1.02	1.25	1.36	1.34	1.33
15–20	0.93	1.05	1.04	1.00	1.12	1.34	1.30	1.29
Trt × depth	0.150				0.875			
	*D_t_* (mm^2^ s^−1^) at saturation							
0–5	0.42 b	0.58 a	0.51 ab	0.55 a	0.67	0.65	0.56	0.61
5–10	0.58	0.53	0.61	0.56	0.65	0.78	0.69	0.76
10–15	0.57	0.52	0.58	0.54	0.64	0.66	0.71	0.68
15–20	0.51	0.55	0.55	0.53	0.61	0.69	0.70	0.69
Trt × depth	0.039				0.734			
	*D_t_* (mm^2^ s^−1^) at PWP							
0–5	0.42 b	0.65 a	0.55 ab	0.47 b	0.70 a	0.57 ab	0.48 b	0.62 ab
5–10	0.62 a	0.59 ab	0.62 a	0.52 b	0.69	0.67	0.71	0.69
10–15	0.57	0.52	0.56	0.56	0.65	0.60	0.71	0.66
15–20	0.54	0.55	0.57	0.52	0.66	0.58	0.70	0.65
Trt × depth	0.052				0.401			

§ Means those sharing lower-case letters within a row are not different at α = 0.05. Abbreviations: Trt., treatment; OWB, Old-World bluestem; TW, tall wheatgrass.

## Data Availability

Not applicable.

## Supplementary Materials

The following supporting information can be downloaded at: https://www.mdpi.com/article/10.3390/plants12071491/s1: Table S1: Description of forage and pasture management practices under different treatments over the years in the east pasture area at New Deal, TX, USA. Pasture treatments include native, Old-World Bluestem (OWB) (OWB, *Bothriochloa bladhii* (Retz) T. Blake)-legume, annual, and alfalfa-tall wheatgrass (TW) (TW, *Thinopyrum ponticum* (Host) Beauv.). Area is for a single replicate. Table S2: Description of forage and pasture management practices under different treatments over the years in the west pasture area at New Deal, TX, USA. Pasture treatments include native, teff, Old World bluestem (OWB) (OWB, *Bothriochloa bladhii* (Retz) T. Blake)-ungrazed, and OWB-grazed. Area is for a single replicate.
