# Supplementary material for: Effect of Long-Term Semiarid Pasture Management on Soil Hydraulic and Thermal Properties
Source: Plants (Basel). 2023 Mar 29;12(7):1491. doi: 10.3390/plants12071491 (PMC10096669; doi:10.3390/plants12071491)
Supplement: Supplementary file 1 [file plants-12-01491-s001.zip › plants-2294543-supplementary.pdf]

Supplementary Tables

Supplementary Tables S1 and S2 summarize the description of forage and pasture management practices under different pasture treatments over the years in the study site.

Table S1. Description of forage and pasture management practices under different treatments over the years in the east pasture area at New Deal, Texas, USA. Pasture treatments include native, Old World Bluestem (OWB) [OWB, *Bothriochloa bladhii* (Retz) T. Blake]-legume, annual, and alfalfa-tall wheatgrass (TW) [TW, *Thinopyrum ponticum* (Host) Beauv.]. Area is for a single replicate

Treatment	Forage and establishment	Management	Grazing
Native	Established: 2008<br><br>Area: 2.3 acres<br><br>Species: mixture of blue grama [*Bouteloua gracilis* (Willd. Ex Kunth) Lag. Ex Griffths], sideoats grama [*Bouteloua curtipendula* (Michx.) Torr], and green sprangletop [*Leptochloa dubia* (Kunth.) Nees]	Subsurface drip irrigated in 2014<br><br>Hayed only in 2009<br><br>3 inches irrigation in 2014<br><br>Fertilization: In 2009 54 lb N, 10 lb P_2O_5, 16 lb Sulfur, 1.5 lb Zinc, 0.5 lb Mn and 0.5 lb Bo per acre was applied	Grazed every summer, but with short grazing duration
OWB-legume	Established: March 2010<br><br>Area: 5.2 acre<br><br>Species: WW-B. Dahl mixed with alfalfa (Medicago sativa L.) and yellow sweet clover (*Melilotus officinalis* L.).<br><br>Interseeded to achieve 20% legume content	Subsurface drip irrigated in each summer<br><br>Reseeded in October 2014 to increase legume content<br><br>Legume was less dense in 1st replicate, relative to 2nd and 3rd replicates<br><br>No N fertilization	Grazed since 2008 except 2011–2013 drought period

	Sweet clover disappeared by 2011 and the alfalfa content was less than 15% in 2013		
Annual	Area: 0.6 acre<br><br>Established: 2013<br><br>Species: annual grasses, sorghum-sudangrass [*Sorghum bicolor* (L.) Moench], wheat (*Triticum aestivum* L.), teff [*Eragrostis tef* (Zucc.) Trotter], and cereal rye (*Secale cereale* L.)<br><br>Sorghum-sudangrass was in 1st replicate	Teff in all blocks since 2014<br><br>Conventional cotton (*Gossipium hirsutum* L.) prior to 2011	Grazed occasionally
Alfalfa-TW	Area: 2.3 acres<br><br>Establishment: 2009 (Jose cultivar)<br><br>Low density TW until summer 2015<br><br>Alfalfa and tall wheatgrass were planted in 2009	Subsurface drip irrigated only in 2014<br><br>Hayed in 2009–2015, each year<br><br>Fertilization: 70 lb acre^{-1} P_2O_5 in 2016	Grazed since 2008, except from 2011 to 2013

Table S2. Description of forage and pasture management practices under different treatments over the years in the west pasture area at New Deal, Texas, USA. Pasture treatments include native, teff, Old World bluestem (OWB) [OWB, *Bothriochloa bladhii* (Retz) T. Blake]-ungrazed, and OWB-grazed. Area is for a single replicate

Pasture treatment	Forage and establishment	Management	Grazing
Native	Area: 11.2 acres<br><br>Establishment: 2002	Continuous cotton prior to 2000<br><br>Dryland	Grazed since 2004, except from 2011 to 2013.

	Species: Mixture of buffalograss [*Buchloe dactyloides* (Nutt.) Engelm], blue grama, sideoats grama, and green sprangletop	One hay cutting in 2003 and 2014<br><br>Fertilization: 30 lb N and 15 lb P_2O_5 per acre was applied in 2004; 26 lb N and 30 lb S per acre was applied in spring 2006; 54 N lb, 10 lb P_2O_5, 16 lbs S, 1.5 lb Zn, 0.5 lb Mn, 0.5 lb B per acre applied in 2009	
Teff	Area: three paddocks of total 13 acres<br><br>Establishment: 2004<br><br>Species: Teff, Foxtail millet [*Setaria italica* (L.) P. Beauv.], wheat (*Triticum aestivum* L.) forage sorghum, Tifton 85 bermudagrass (*Cynodon dactylon* L.), and cotton<br><br>No crop in 2011, then Elbon Rye (*Secale* L.) planted in 2012 winter with teff only from 2013 to 2016<br><br>Foxtail millet [*Setaria italica* (L.) P. Beauv.] (2004 – 2008), forage sorghum (2004 and 2008), and cotton rotation before 2011; bermudagrass-teff rotation from 2005 to 2010<br><br>Teff grown since 2013 after killing bermudagrass. Wheat (TAM 112) planted in 2012 as winter cover in one of the paddocks	Subsurface drip irrigation in two paddocks<br><br>Low irrigation treatment from 2009 to 2011. One pasture within this treatment had no irrigation system.<br><br>Part of the trial was completely dryland from 2004 to 2008 and irrigated<br><br>Fertilization: 60 lbs N per year when in foxtail millet and from 30 to 80 lbs N+S and 10-30 lbs P_2O_5 Phosphorus/acre as needed each year<br><br>Teff hayed in 2013, 2014, and 2015<br><br>Left fallow in 2016 for a new trial	Grazed every year from 2005 to 2010 and hayed excess forage mass<br><br>Grazed in 2014 and 2015 in combination with hay.
OWB-ungrazed	Area: 5.1 acres<br><br>Establishment: 2003<br><br>Species: WW-B.Dahl	Subsurface drip irrigation<br><br>Hayed from 2013 to 2015	Grazed every year since 2005 except 2011, 2012, and 2013

		Harvested for seed in 2006, 2007, 2008, 2010, 2013, and 2014.<br><br>Fertilization: 60 lb N $acre^{-1}$ + S	
OWB-grazed	Same as OWB-ungrazed	Same as OWB-ungrazed	No grazing, only hayed in 2014, 2015, and 2016
